# Association of the high-density lipoprotein cholesterol to C-reactive protein ratio with chronic cough in US adults: Effect modification by smoking status

**DOI:** 10.18332/tid/222393

**Published:** 2026-07-26

**Authors:** Xuefeng Li, Jian Zhang, Can Wang, Yulu Yang, Qiang Wang, Xuefei Bai, Yi Tang, Meiling Zhang

**Affiliations:** 1 Department of Thoracic Oncology, Hefei Cancer Hospital, Chinese Academy of Sciences, Hefei, China; 2 Department of Clinical Laboratory, Changzhou Maternal and Child Health Care Hospital, Changzhou, China; 3 Department of Respiratory, Fuyang Hospital of Anhui Medical University, Fuyang, China; 4 Department of Obstetrics and Gynecology, The First Affiliated Hospital of Anhui Medical University, Hefei, China; 5 Department of Respiratory, Fuyang People's Hospital, Fuyang, China; 6 Department of Respiratory, The Second People’s Hospital of Wuhu, East China Normal University, Wuhu, China

**Keywords:** HDL-C/CRP ratio, chronic cough, NHANES, cross-sectional study, smoking status

## Abstract

**INTRODUCTION:**

Systemic inflammation may mediate the association between chronic cough and lipid metabolism. The high-density lipoprotein cholesterol to C-reactive protein (HDL-C/CRP) ratio, a composite marker of metabolic-inflammatory balance, has not been evaluated in this context. Smoking impairs high-density lipoprotein (HDL) function and elevates CRP, yet the combined effect of these pathways on chronic cough remains unknown. This study examined the association between the HDL-C/CRP ratio and chronic cough in US adults, and assessed whether smoking status modified this association.

**METHODS:**

This secondary analysis study used cross-sectional data from the 2005–2010 National Health and Nutrition Examination Survey (NHANES). Chronic cough was defined as a recurrent cough on most days for ≥3 consecutive months per year (self-reported). Serum HDL-C and CRP were measured by standardized assays. Multivariate logistic regression was employed to assess the association between the natural logarithm of the ratio of HDL-C to CRP [Ln (HDL-C/CRP)] and chronic cough. Subgroup analyses examined the interaction with smoking status, and sensitivity analyses validated the robustness of our findings. Nonlinear relationships were assessed using restricted cubic splines (RCS).

**RESULTS:**

The study enrolled 6762 participants, 672 of whom had chronic cough. After full adjustment, each unit increase in the Ln (HDL-C/CRP) was associated with a 13% lower risk of chronic cough (AOR=0.87; 95% CI: 0.78–0.97). RCS showed a linear relationship and negative correlation between Ln (HDL-C/CRP) and chronic cough (p for nonlinearity=0.779, p for overall=0.021). Subgroup analysis showed the association varied across smoking status groups (interaction p=0.005), with the most pronounced association observed among never smokers (OR=0.735; 95% CI: 0.602–0.898).

**CONCLUSIONS:**

This cross-sectional study demonstrates that a higher HDL-C/CRP ratio is associated with a lower prevalence of chronic cough, exhibiting a linear dose–response relationship. Notably, this association is most pronounced in never smokers, suggesting a potential modifying effect of smoking status.

## INTRODUCTION

Chronic cough, a frequently encountered symptom in clinical practice, is clinically defined in adults as a cough that persists for more than 8 weeks^1,2^. The global prevalence of chronic cough varies significantly by region, ranging from 2–18% overall, as reported in a recent systematic review and meta-analysis^3^. The affected population is primarily middle-aged individuals, with a higher prevalence observed in females compared to males^4^. However, this gender and age effect may vary across different regions, environments, or social contexts^5,6^. In addition, chronic cough imposes a heavy health burden on patients. On the one hand, frequent medical visits and treatments lead to increased financial costs and excessive utilization of healthcare resources. On the other hand, chronic cough often results in comorbidities and concomitant symptoms, reduces lung function, and negatively affects quality of life^7^. Therefore, identifying novel risk factors for chronic cough is crucial for optimizing preventive measures, risk stratification, and improving patient management.

Chronic cough is characterized by neutrophilic airway inflammation, which drives airway remodeling, hyperresponsiveness, and neurogenic inflammation^8^. C-reactive protein (CRP), an acute-phase reactant primarily synthesized by the liver, is not only a recognized marker of systemic inflammation but also implicated in the pathophysiology of various lung diseases^9^. Landt et al.^10^ showed that in chronic obstructive pulmonary disease (COPD), comorbid chronic cough is associated with elevated serum high-sensitivity CRP levels. Whether this association extends to the general population and whether CRP alone optimally reflects the inflammatory burden of chronic cough remains unclear.

A positive association between the non-high-density lipoprotein to high-density lipoprotein cholesterol ratio (NHHR) and chronic cough has been reported, with systemic inflammation partially mediating this relationship^11^. However, the NHHR primarily reflects atherogenic lipid burden. High-density lipoprotein cholesterol (HDL-C) is commonly used to clinically assess high-density lipoprotein (HDL) metabolism^12^. HDL has been shown to possess anti-inflammatory and antioxidant properties and to counteract CRP-induced pro-inflammatory effects in vitro^13^. Although HDL-C does not fully reflect HDL function, low HDL-C levels are independently associated with accelerated decline in lung function and are implicated in various inflammatory airway diseases^14-16^. Given that HDL-C is a key indicator of HDL levels, the association between HDL-C and CRP may reflect systemic inflammation and hold clinical significance.

In chronic airway diseases, the HDL-C/CRP ratio has demonstrated significant clinical value. Specifically, in idiopathic pulmonary fibrosis (IPF), this ratio acts as an independent protective factor, showing superior predictive efficacy compared to HDL-C or CRP alone^17^. However, to date, no studies have specifically considered the association between the HDL-C/CRP ratio and chronic cough. This study represents the first examination of this association in a general population. Furthermore, smoking, a significant environmental exposure, has been well-documented to simultaneously impair HDL functionality (as measured by cholesterol efflux capacity and anti-inflammatory properties) and elevate systemic inflammation (as indicated by increased CRP levels)^18,19^.

Whether smoking-related changes in HDL and CRP collectively contribute to the risk of respiratory conditions, such as chronic cough, requires further investigation. Based on the interplay between systemic inflammation (represented by CRP), lipid metabolism (represented by HDL-C), and the known impact of smoking on both pathways, it was hypothesized that: 1) a lower HDL-C/CRP ratio is associated with a higher prevalence of chronic cough; and 2) smoking status may modify this association.

## METHODS

### Study design and population

**Figure 1:**
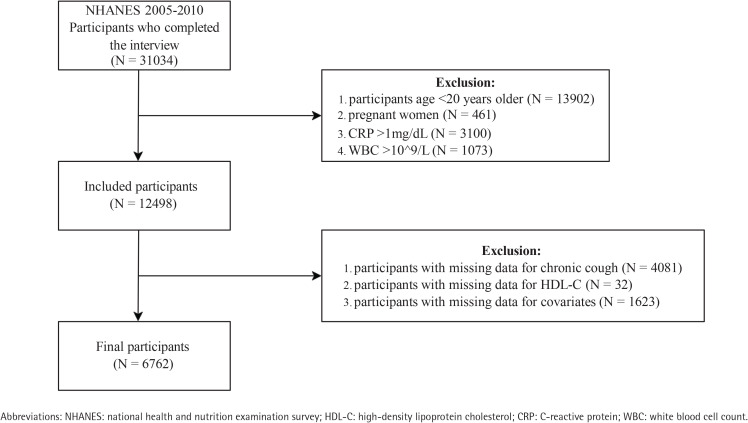
Flow chart of participant selection

This secondary analysis study used cross-sectional data from the 2005–2010 National Health and Nutrition Examination Survey (NHANES). NHANES is a research project designed to assess the nutrition and overall health of the US population. The survey is divided into two main parts: physical examination, and interview. All participants volunteered to participate in the study and signed an informed consent form. Data were extracted from the NHANES cycles 2005–2010, which employed a complex stratified multistage probability sampling design to obtain a nationally representative sample of the non-institutionalized civilian US population. The initial sample size was 31034 participants. Of these, 24272 participants were excluded for various reasons: age <20 years (n=13902), pregnancy (n=461), CRP >1 mg/dL (n=3100), white blood cell count (WBC) >10×10^9^/L (n=1073), missing data on chronic cough (n=4081), missing data on HDL-C (n=32), and lack of covariate data (n=1623). Consequently, a total of 6762 participants were included in the final analysis. The detailed inclusion and exclusion process is shown in Figure 1.

### Assessment of HDL-C /CRP ratio

**Figure 2:**
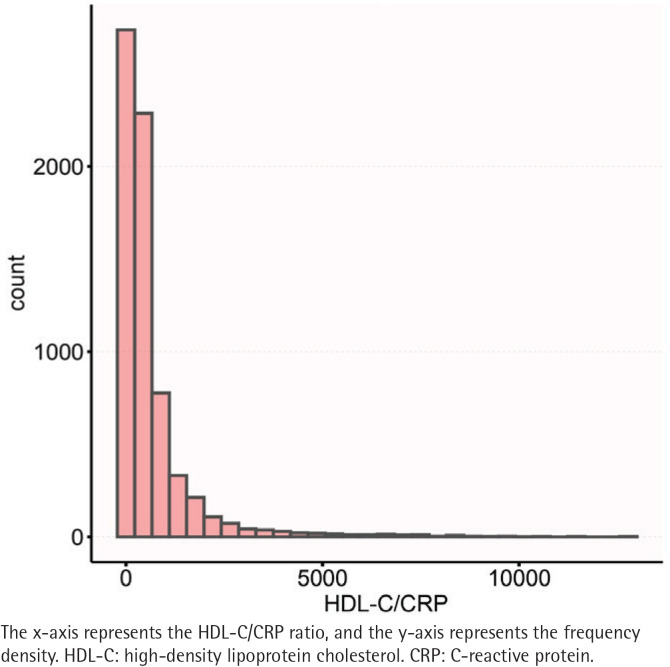
Histogram of the distribution of HDL-C/CRP ratio

CRP was measured by the latex-enhanced turbidity method, where CRP in the sample binds to anti-CRP antibodies on latex particles, causing turbidity changes that were used to quantify CRP. Consistent with recommendations in the literature, participants with serum CRP levels above 1 mg/dL were excluded from the analysis to mitigate potential biases arising from acute infection-induced elevations in CRP^20^. Additionally, individuals with WBC counts >10×10^9^/L were excluded to rule out acute infections. HDL-C was measured by chemically isolating non-HDL-C and then using an enzymatic reaction to convert HDL-C into a detectable pigment, the concentration of which was then measured by a photometer. The independent variable was defined as the HDL-C/CRP ratio. The distribution of this ratio was right-skewed, as indicated by the histogram (Figure 2).

### Assessment of chronic cough

The dependent variable in this study was chronic cough, which was determined by means of a questionnaire. Specifically, participants were asked the following question by a medical professional: ‘Do you often have a cough that lasts for three consecutive months or longer each year on most days?’. When participants answered ‘yes’, they were identified as having a chronic cough^11^. This definition differs from the clinical guideline definition of >8 weeks^21^; this discrepancy is attributable to the fixed questionnaire design of large national databases rather than investigator preference.

### Assessment of covariates

In this study, the selection of covariates was informed by previous published literature and included the following variables: age, gender (male, female; based on self-report), race (Non-Hispanic White, Non-Hispanic Black, Mexican American, and Other), body mass index (kg/m^2^), education level, marital status, poverty income ratio (PIR), smoking status, drinking status, total cholesterol level, diabetes, asthma, emphysema, chronic bronchitis, cancer, heart failure, coronary artery disease, hypertension, infections of pneumonia and influenza within the past 30 days (P&I-30d), blood eosinophil count (BEC), WBC, and stroke^11,22^.

Diagnoses of respiratory diseases (asthma, chronic bronchitis, and emphysema), cardiovascular diseases (hypertension, coronary heart disease, and heart failure), cancer, stroke, P&I-30d, and endocrine diseases (diabetes) were based on self-reported data collected via questionnaires in the NHANES database. For example, in the questionnaire, a ‘yes’ response to the question of whether one has chronic bronchitis indicates the presence of the disease, while a ‘no’ response indicates its absence. The drinking covariate was defined as follows: participants who had consumed <12 alcoholic drinks in their lifetime were categorized as non-drinkers; conversely, participants who had consumed ≥12 alcoholic drinks were categorized as drinkers. The marital status of the participants was categorized into two groups: one group was married/living with a partner, and the other group was living alone, which included those who were widowed, divorced, separated, or never married. We assessed the impact of household income by categorizing the poverty income ratio (PIR) into three groups: <1.3, 1.3–3.5, and >3.5. Education level was categorized into three groups: lower than high school, high school or equivalent, and college graduate or higher. Smoking status was categorized into three groups: those who had smoked <100 cigarettes in their lifetime (never smokers), those who had smoked ≥100 cigarettes but did not currently smoke (former smokers), and those who had smoked ≥100 cigarettes and continue to smoke (including occasional or daily smokers) (current smokers)^23^.

### Statistical analysis

All statistical analyses were conducted using R version 4.4.2 (https://www.r-project.org/) and Free Statistics software version 2.0^24^. Statistical significance was set at p<0.05. All analyses incorporated sampling weights adjusted for multiple NHANES cycles. For the combined 2005–2010 dataset, 2-year MEC examination weights were divided by the number of included cycles (n=3) to maintain appropriate population estimates, following NCHS recommendations for pooled NHANES analyses. Complex survey design was accounted for using adjusted weights, primary sampling units (SDMVPSU), and strata (SDMVSTRA). Continuous variables with normal distribution are described as mean ± standard deviation; non-normally distributed variables are described as median (interquartile range). Categorical variables are presented as frequencies and percentages. Multicollinearity was assessed using generalized variance inflation factors (GVIF) and Spearman’s rank correlation coefficients. Variables exhibiting significant multicollinearity were identified based on the criterion GVIF^(1/2df)^ >2.5, where df denotes degrees of freedom. Given that the natural logarithm (Ln) or common logarithm (log) transformation is often used in the previous literature for skewed distribution of ratio-type independent variables, the Ln transformation was performed for HDL-C/CRP ratio in this study^25,26^. Ln (HDL-C/CRP) was analyzed as a continuous variable (primary analysis) and categorized into tertiles to explore potential threshold effects. The multivariate logistic regression model was used to investigate the association between CRP, HDL-C, and Ln (HDL-C/CRP) with chronic cough across different models. Specifically, Ln (HDL-C/CRP) was analyzed both as a continuous variable and in tertiles, while CRP and HDL-C were analyzed solely as continuous variables in the multivariate logistic regression analysis. Covariates were adjusted in three models: Model 1 included no covariate adjustments; Model 2 adjusted for age, gender, and race; Model 3 adjusted as for Model 2 plus body mass index (BMI), education level, marital status, PIR, smoking status, drinking status, diabetes, asthma, emphysema, chronic bronchitis, cancer, heart failure, coronary artery disease, hypertension, stroke, P&I-30d, WBC, BEC, and total cholesterol. In addition, the relationship between Ln (HDL-C/CRP) (continuous) and chronic cough was assessed using restricted cubic splines (RCS), with the stability of this relationship further evaluated via subgroup analyses. Sensitivity analyses were additionally performed using quartiles after excluding participants with respiratory comorbidities (asthma, chronic bronchitis, or emphysema), despite the reduced sample size.

## RESULTS

### Population characteristics

This study included 6762 unweighted participants, of whom 672 had chronic cough. Table 1 presents the baseline characteristics of the study population. The weighted mean age of the overall sample was 56.84 ± 11.84 years, with 49.46% being male. In terms of race, the majority were non-Hispanic White (77.51%), followed by non-Hispanic Black (9.05%), Mexican-American (5.71%), and other races (7.73%). Individuals with chronic cough were older and more likely to be non-Hispanic White than those without chronic cough. Significant differences (p<0.05) were observed between the chronic cough group and the non-chronic cough group across multiple variables. These variables included demographic characteristics (age, race, education level, and PIR), lifestyle factors (smoking status), respiratory diseases (asthma, chronic bronchitis, and emphysema), cardiovascular diseases (heart failure and hypertension), stroke, P&I-30d, and biomarkers [Ln (HDL-C/CRP), BEC, WBC, and CRP]. No significant differences were observed for gender, BMI, total cholesterol, HDL-C, cancer, marital status, drinking status, or coronary artery disease.

**Table 1:** Basic characteristics of NHANES participants stratified by chronic cough status (weighted N=88946668)

*Characteristics*	*Overall weighted* *n (%)*	*No chronic cough weighted* *n (%)*	*Chronic cough weighted* *n (%)*	*p*
**Total**, n	88946668	80007025	8939643	
**Age** (years), mean (SD)	56.84 (11.84)	56.69 (11.82)	58.20 (11.94)	**0.01**
**Gender**				0.684
Male	43994547 (49.46)	39669806 (49.58)	4324741 (48.38)	
Female	44952121 (50.53)	40337219 (50.42)	4614902 (51.62)	
**Race**				0.001
Non-Hispanic White	68947996 (77.51)	61392291 (76.73)	7555705 (84.52)	
Non-Hispanic Black	8046640 (9.05)	7406753 (9.26)	639887 (7.16)	
Mexican-American	5075450 (5.71)	4735805 (5.92)	339645 (3.80)	
Other	6876582 (7.73)	6472176 (8.09)	404406 (4.52)	
**Smoking status**				**<0.001**
Never smoker	46507121 (52.29)	43637678 (54.54)	2869443 (32.10)	
Former smoker	27949337 (31.42)	25650768 (32.06)	2298569 (25.71)	
Current smoker	14490210 (16.29)	10718579 (13.40)	3771631 (42.19)	
**Education level**				**<0.001**
Lower than high school	5699441 (6.41)	4944020 (6.18)	755422 (8.45)	
Hight school or equivalent	31780863 (35.73)	27607613 (34.51)	4173250 (46.68)	
College graduate or higher	51466364 (57.86)	47455392 (59.31)	4010971 (44.87)	
**Marital status**				**0.051**
Married/living with partner	63091935 (70.93)	57179651 (71.47)	5912285 (66.14)	
Living alone	25854733 (29.07)	22827374 (28.53)	3027358 (33.86)	
**PIR**				**0.001**
<1.3 low	12902771 (14.51)	11137012 (13.92)	1765759 (19.75)	
1.3–3.5 medium	30383631 (34.16)	27046700 (33.81)	3336931 (37.33)	
>3.5 high	45660266 (51.33)	41823313 (52.27)	3836953 (42.92)	
**Drinking status**				0.097
No	23107708 (25.98)	21010613 (26.26)	2097095 (23.46)	
Yes	65838960 (74.02)	58996412 (73.74)	6842548 (76.54)	
**Asthma**				**<0.001**
No	78496274 (88.25)	71934307 (89.91)	6561967 (73.40)	
Yes	10450394 (11.75)	8072718 (10.09)	2377676 (26.60)	
**Emphysema**				**<0.001**
No	86992856 (97.80)	78961670 (98.69)	8031185 (89.84)	
Yes	1953812 (2.20)	1045355 (1.31)	908458 (10.16)	
**Chronic bronchitis**				**<0.001**
No	83480259 (93.85)	76397902 (95.49)	7082357 (79.22)	
Yes	5466409 (6.15)	3609123 (4.51)	1857285 (20.78)	
**Heart failure**				**<0.001**
No	86531647 (97.28)	78172133 (97.71)	8359514 (93.51)	
Yes	2415021 (2.72)	1834892 (2.29)	580129 (6.49)	
**Coronary artery disease**				0.133
No	84670543 (95.19)	76284647 (95.35)	8385896 (93.80)	
Yes	4276125 (4.81)	3722378 (4.65)	553747 (6.20)	
**Hypertension**				**0.003**
No	59316101 (66.69)	53909196 (67.39)	5406906 (60.48)	
Yes	29630567 (33.31)	26097829 (32.61)	3532737 (39.52)	
**Diabetes**				**0.024**
No	79752333 (89.66)	71946778 (89.93)	7805555 (87.31)	
Yes	9194335 (10.34)	8060247 (10.07)	1134088 (12.69)	
**Cancer**				0.532
No	77683543 (87.34)	69956492 (87.44)	7727051 (86.44)	
Yes	11263125 (12.66)	10050533 (12.56)	1212592 (13.56)	
**Stroke**				**<0.001**
No	85836740 (96.50)	77468801 (96.83)	8367939 (93.60)	
Yes	3109928 (3.50)	2538224 (3.17)	571704 (6.40)	
**Pɛtl-30d**				**<0.001**
No	85865783 (96.54)	77549853 (96.93)	8315929 (93.02)	
Yes	3080885 (3.46)	2457172 (3.07)	623714 (6.98)	
**BMI** (kg/m^2^), mean (SD)	28.46 (5.95)	28.45 (5.85)	28.58 (6.79)	0.723
**Total cholesterol** (mg/dL), mean (SD)	203.51 (41.46)	203.73 (41.42)	201.56 (41.78)	0.251
**HDL-C** (mg/dL), mean (SD)	54.92 (16.66)	55.06 (16.58)	53.66 (17.38)	0.064
**CRP** (mg/dL), median (IQR)	0.16 (0.07–0.33)	0.16 (0.07–0.32)	0.21 (0.09–0.41)	**<0.001**
**BEC** (109/L), median (IQR)	0.20 (0.10–0.20)	0.20 (0.10–0.20)	0.20 (0.10–0.30)	**<0.001**
**WBC** (109/L), median (IQR)	6.60 (5.60–7.80)	6.50 (5.50–7.70)	7.10 (5.90–8.10)	**<0.001**
**Ln** (HDL-C/CRP), mean (SD)	5.87 (1.14)	5.90 (1.14)	5.59 (1.09)	**<0.001**
**Tertile of Ln** (HDL-C/CRP)				**<0.001**
T1	26717944 (30.04)	23140442 (28.92)	3577503 (40.02)	
T2	29263089 (32.90)	26539527 (33.17)	2723562 (30.47)	
T3	32965635 (37.06)	30327056 (37.91)	2638578 (29.51)	

Ln (HDL-C/CRP): natural logarithm of the ratio of high-density lipoprotein cholesterol to C-reactive protein. BMI: body mass index. PIR: poverty income ratio. Pɛtl-30d: infections of pneumonia and influenza within the past 30 days. WBC: white blood cell count. BEC: blood eosinophil count. T: tertile. Ln (HDL-C/CRP) tertile ranges: T1, 3.05–5.18; T2, 5.18–6.21; T3, 6.21–9.46. Weighted analyses were performed to represent the general population. Data are presented as weighted mean (SD), weighted median (IQR), or weighted n (%). Bold values indicate statistical significance.

### Association between HDL-C/CRP ratio and chronic cough

**Figure 3:**
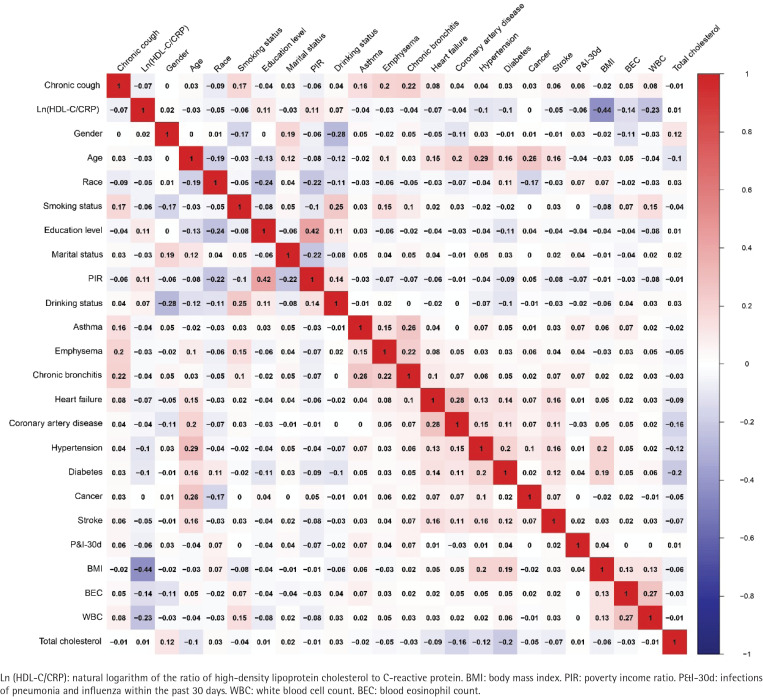
Heatmap of Spearman’s rank correlation coefficients

Prior to conducting the multivariate logistic regression analysis, we assessed multicollinearity among all candidate variables, encompassing both categorical and continuous variables, to ensure the stability and reliability of model estimates. All GVIF^(1/2df)^ values were <2.5, and all absolute pairwise Spearman’s rank correlation coefficients were <0.7, indicating no severe multicollinearity (Table 2, Figure 3).

**Table 2:** Variance inflation factors for variables

*Variable*	*GVIF*	*df*	*GVIF^(1/2df)^*
Gender	1.272	1	1.102
Age (years)	1.552	1	1.246
Race	1.488	3	1.069
Smoking status	1.525	2	1.111
Education level	1.469	2	1.101
Marital status	1.152	1	1.073
PIR	1.377	2	1.083
Drinking status	1.206	1	1.098
Asthma	1.139	1	1.067
Emphysema	1.116	1	1.056
Chronic bronchitis	1.138	1	1.067
Heart failure	1.193	1	1.092
Coronary artery disease	1.186	1	1.089
Hypertension	1.217	1	1.103
Diabetes	1.205	1	1.098
Cancer	1.110	1	1.053
Stroke	1.090	1	1.044
Pɛtl-30d	1.035	1	1.018
BMI	1.384	1	1.177
WBC	1.194	1	1.093
BEC	1.101	1	1.049
Total cholesterol	1.107	1	1.052

BMI: body mass index. PIR: poverty income ratio. Pɛtl-30d: infections of pneumonia and influenza within the past 30 days. WBC: white blood cell count. BEC: blood eosinophil count. GVIF: Generalized Variance Inflation Factor. df: degrees of freedom.

In the fully adjusted model (Model 3), each one-unit increment in Ln (HDL-C/CRP) was associated with a 13% lower risk of chronic cough when Ln (HDL-C/CRP) was analyzed as a continuous variable (AOR=0.87; 95% CI: 0.78–0.97; p=0.015). In contrast, for every one-unit increase in CRP, the risk of chronic cough increased by 71% (AOR=1.71; 95% CI: 1.05–2.77; p=0.033). HDL-C alone showed a negligible, non-significant association with chronic cough risk (AOR=0.999 per 1 mg/dL, 95% CI 0.991–1.007; p=0.814) (Table 3). When Ln (HDL-C/CRP) was divided into tertiles, there was a non-significant inverse trend (p for trend=0.080). Compared to T1, T2 showed a non-significant 18% lower risk of chronic cough (AOR=0.82; 95% CI: 0.65–1.03; p=0.090), and T3 showed a non-significant 24% lower risk of chronic cough (AOR=0.76; 95% CI: 0.56–1.04; p=0.085) (Table 3).

**Table 3:** Association of CRP, HDL-C, and Ln (HDL-C/CRP) with chronic cough in weighted multivariable logistic regression models

*Variables*		*Model 1*		*Model 2*		*Model 3*	
		*OR (95% CI)*	*p*	*AOR (95% CI)*	*p*	*AOR (95% CI)*	*p*
**CRP**	continuous	2.76 (1.90–4.01)	**<0.001**	2.82 (1.93–4.11)	**<0.001**	1.71 (1.05–2.77)	**0.033**
**HDL-C**	continuous	0.995 (0.989–1.001)	0.076	0.993 (0.985–1.0003)	0.060	0.999 (0.991–1.007)	0.814
**Ln** (HDL-C/CRP)	continuous	0.78 (0.71–0.86)	**<0.001**	0.77 (0.70–0.86)	**<0.001**	0.87 (0.78–0.97)	**<0.015**
	T1 (ref.)	1		1		1	
	T2	0.66 (0.55–0.80)	**<0.001**	0.66 (0.55–0.79)	**<0.001**	0.82 (0.65–1.03)	0.090
	T3	0.56 (0.43–0.74)	**<0.001**	0.56 (0.42–0.73)	**<0.001**	0.76 (0.56–1.04)	0.085
	p for trend		**<0.001**		**<0.001**		0.080

Ln (HDL-C/CRP): natural logarithm of the ratio of high-density lipoprotein cholesterol to C-reactive protein. BMI: body mass index. PIR: poverty income ratio. Pɛtl-30d: infections of pneumonia and influenza within the past 30 days. WBC: white blood cell count. BEC: blood eosinophil count. T: tertile. Ln (HDL-C/CRP) tertile ranges: T1, 3.05–5.18; T2, 5.18–6.21; T3, 6.21–9.46. Model 1: no covariates were adjusted. AOR: adjusted odds ratio. Model 2: adjusted for age, gender and race. Model 3: adjusted as for Model 2 plus BMI, education level, marital status, PIR, smoking status, drinking status, diabetes, asthma, emphysema, chronic bronchitis, cancer, heart failure, coronary artery disease, hypertension, stroke, Pɛtl-30d, WBC, BEC and total cholesterol.

### Restricted cubic splines

RCS analysis showed a significant linear association between Ln (HDL-C/CRP) and chronic cough (p for overall=0.021; p for nonlinearity=0.779) (Supplementary file Figure 1).

### Subgroup analysis and sensitivity analysis

**Figure 4:**
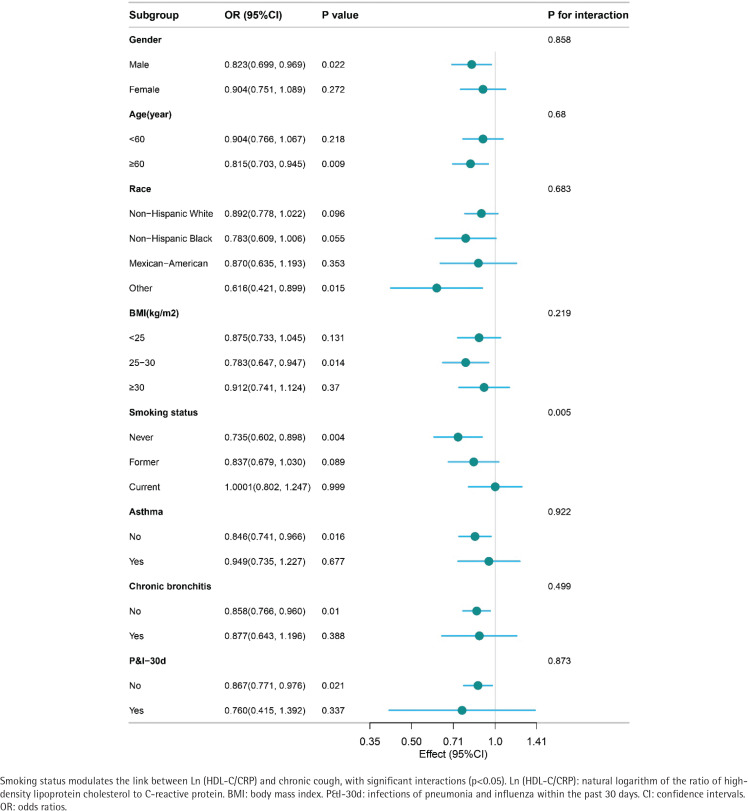
Weighted subgroup analyses to examine the association between Ln (HDL-C/CRP) and chronic cough across strata of gender, age, race, BMI, smoking status, asthma, chronic bronchitis, and P&I-30d

Subgroup analyses were performed according to gender, age groups (<60 years, ≥60 years), race, BMI (kg/m^2^) groups (<25, 25–30, ≥30), smoking status, asthma, chronic bronchitis, and P&I-30d. The results indicated a significant interaction between Ln (HDL-C/CRP) and chronic cough in the subgroup of smoking status (p for interaction=0.005). No significant interactions were detected in the remaining subgroups (p for interaction >0.05) (Figure 4). In a sensitivity analysis excluding participants with asthma, emphysema, or chronic bronchitis, Ln (HDL-C/CRP) was analyzed in quartiles. Compared to Q1, the adjusted ORs for Q2, Q3, and Q4 were 0.694 (95% CI: 0.475–1.016; p=0.059), 0.700 (95% CI: 0.490–0.9994; p=0.0496), and 0.604 (95% CI: 0.384–0.948; p=0.030), respectively. A significant linear trend was observed (p for trend=0.032) (Table 4).

**Table 4:** Association of Ln (HDL-C/CRP) with chronic cough in weighted multivariable logistic regression models after exclusion of participants with asthma, emphysema, or chronic bronchitis

*Variable*		*Model 1*		*Model 2*		*Model 3*	
		*OR (95% CI)*	*p*	*AOR (95% CI)*	*p*	*AOR (95% CI)*	*p*
**Ln** (HDL-C/CRP)	continuous	0.80 (0.72–0.89)	**<0.001**	0.8 (0.72–0.89)	**<0.001**	0.85 (0.75–0.96)	**0.012**
	Q1 (ref.)	1		1		1	
	Q2	0.71 (0.49–1.03)	0.069	0.70 (0.48–1.01)	0.056	0.694 (0.475–1.016)	0.059
	Q3	0.61 (0.44–0.84)	**0.003**	0.59 (0.44–0.81)	**0.001**	0.700 (0.490–0.9994)	**0.0496**
	Q4	0.52 (0.36–0.76)	**0.001**	0.51 (0.35–0.76)	**0.001**	0.604 (0.384–0.948)	**0.030**
	p for trend		**<0.001**		**<0.001**		**0.032**

Ln (HDL-C/CRP): natural logarithm of the ratio of high-density lipoprotein cholesterol to C-reactive protein. BMI: body mass index. PIR: poverty income ratio. Pɛtl-30d: infections of pneumonia and influenza within the past 30 days. WBC: white blood cell count. BEC: blood eosinophil count. Q: quartile. Ln (HDL-C/CRP) quartile ranges: Q1, 3.23–4.92; Q2, 4.92–5.72; Q3, 5.72–6.55; Q4, 6.55–9.46. Model 1: no covariates were adjusted. AOR: adjusted odds ratio. Model 2: adjusted for age, gender and race. Model 3: adjusted as for Model 2 plus BMI, education level, marital status, PIR, smoking status, drinking status, diabetes, cancer, coronary artery disease, hypertension, WBC, BEC and total cholesterol and stroke.

## DISCUSSION

The cross-sectional study demonstrated that higher Ln (HDL-C/CRP) levels were significantly associated with a reduced risk of chronic cough, exhibiting a significant linear association. When the individual components were examined in the same multivariable-adjusted model, CRP was independently and positively associated with cough risk, whereas HDL-C showed no independent association. This suggests that the HDL-C/CRP ratio, as an integrated inflammatory-metabolic indicator, may capture the interplay between anti-inflammatory and pro-inflammatory states that is not fully reflected by either marker alone. Notably, the association between Ln (HDL-C/CRP) and chronic cough risk was significantly modified by smoking status.

While the primary weighted analysis showed a non-significant inverse trend, this likely reflects confounding by pre-existing respiratory conditions that independently elevate systemic inflammation and cough risk, thereby diluting the protective association in the broader population. The potential inverse association was further explored by a sensitivity analysis excluding participants with asthma, emphysema, or chronic bronchitis, in which a significant linear trend across quartiles was observed. This pattern is consistent with subgroup analyses showing that the significant protective association was confined to individuals without these respiratory comorbidities.

This cross-sectional study revealed that a higher Ln (HDL-C/CRP) was linearly associated with a lower risk of chronic cough. Deconstructing this ratio, we found that CRP was independently and positively associated with cough risk, whereas HDL-C showed no significant association after multivariable adjustment. This pattern suggests a dynamic equilibrium between pro-inflammatory and protective lipid pathways. The HDL-C/CRP ratio, as a composite measure, integrates this balance, providing a more comprehensive representation of the underlying metabolic-inflammatory interplay in chronic cough than individual markers alone. This finding aligns with the results in an IPF cohort reported by Ouyang et al.^17^ who confirmed that the HDL-C/CRP ratio is an independent protective factor for all-cause mortality or lung transplantation. Furthermore, the negative correlation observed in our study between the HDL-C/CRP ratio and chronic cough contrasts with the positive association for NHHR reported by Wang et al.^11^. This divergence highlights a fundamental distinction between composite indicator types. Unlike previous studies that primarily focused on ‘lipid-lipid’ composites (e.g. NHHR), our research introduces a ‘lipid-inflammation’ composite dimension (HDL-C/CRP), thereby providing a novel framework for understanding the systemic metabolic-inflammatory interactions in chronic cough. As an exploratory mechanistic analysis, this study aimed to elucidate the link between systemic anti-inflammatory/pro-inflammatory balance and chronic cough. Future large-scale longitudinal studies are necessary to validate these associations and assess their clinical applicability.

Subgroup analyses in this cross-sectional study revealed that smoking status significantly modified the association between the HDL-C/CRP ratio and the prevalence of chronic cough. A stepwise disappearance of the inverse association was observed: the association was strongest among never smokers, borderline significant among former smokers, and completely abolished among current smokers. This pattern persisted after multivariate adjustment, implying that underlying biological mechanisms may underpin this modification effect. It is hypothesized that the observed gradient effect is attributable to the disruptive impact of smoking on the lipoprotein-inflammatory axis. First, smoking is a key driver of systemic inflammatory burden. Evidence indicates that smokers exhibit significantly elevated serum high-sensitivity CRP levels^19^. This alone may compromise the assessment efficacy of ratios using CRP as the denominator. Moreover, smoking directly impairs the functional integrity of HDL through oxidative stress and lipid peroxidation pathways, reducing its cholesterol efflux capacity and antioxidant activity^27^. This dysfunction transforms HDL from an anti-inflammatory carrier into an inactive particle, potentially disrupting the HDL-C/CRP ratio’s ability to reflect true anti-inflammatory status, as suggested by studies on HDL dysfunction^12,18^. The intermediate effects observed after smoking cessation can be attributed to the alleviation of oxidative stress and the gradual restoration of HDL function^27^. These findings suggest that smoking may influence respiratory health by disrupting the lipid-inflammation axis. The complete loss of protective association among current smokers strongly supports establishing smoking cessation as a cornerstone intervention for modulating this axis.

However, these findings appear to contrast with prior evidence. The smoking-stratified gradient observed – strongest protection in never smokers and lost in current smokers – differs from the result by Lim et al.^28^ that CRP predicted COPD primarily in ever smokers. This discrepancy is biologically plausible: CRP rises with smoking-induced inflammation, thereby strengthening its association with airway disease among smokers; conversely, the anti-inflammatory effects of HDL-C may be masked by the oxidative burden of smoking. Whether this interaction replicates in other cohorts remains to be determined.

The mechanism underlying the relationship between the HDL-C/CRP ratio and chronic cough remains unclear. However, the following three points validate the plausibility of our research findings that an increased HDL-C/CRP ratio is associated with a reduced risk of chronic cough.

First, elevated systemic inflammation characterized by increased CRP has been observed in chronic respiratory diseases. Landt et al.^10^ demonstrated that individuals with COPD and comorbid chronic cough exhibited significantly higher levels of high-sensitivity CRP, fibrinogen, and circulating neutrophils compared to those with COPD but without chronic cough, suggesting that systemic inflammatory burden is associated with a more severe disease phenotype. In stable COPD, serum CRP levels show a weak but significant positive correlation with the cough component of the COPD assessment test^29^. In non-allergic asthma, hs-CRP levels are higher than in non-asthmatic subjects and in those with allergic asthma, and are associated with respiratory symptoms such as nocturnal cough^30^. These cross-sectional observations suggest an association between systemic inflammatory markers and cough symptoms in chronic airway diseases.

Second, vascular biology research has confirmed that HDL can neutralize CRP-induced endothelial inflammatory responses through its oxidized phospholipid components (particularly oxidized phosphatidylcholine, oxPLPC), significantly inhibiting the expression of adhesion molecules such as vascular cell adhesion molecule-1 (VCAM-1), intercellular adhesion molecule-1 (ICAM-1), and E-selectin^13^. Importantly, HDL particles are not confined to the systemic circulation; emerging evidence indicates that HDL plays specific roles in pulmonary biology, including modulation of cholesterol homeostasis in alveolar macrophages, regulation of surfactant metabolism, and participation in innate immune defense in the lung via apolipoprotein A-I and ABC transporters (ABCA1/G1)^14^. Collectively, these lung-specific functions^14,31^ and HDL’s well-documented extra-cardiovascular roles^32^ indicate that the anti-inflammatory activity of HDL may extend beyond the vascular endothelium to the pulmonary microenvironment.

Third, VCAM-1 and ICAM-1 are not only involved in cardiovascular inflammation but also play crucial roles in respiratory diseases^33^. Specifically, they mediate the transendothelial migration of leukocytes into airway tissues^33^. Neutrophils, which are key effector cells in chronic cough airway inflammation, exhibit infiltration levels that are closely correlated with cough frequency and airway hyperresponsiveness^8^.

These considerations support the biological plausibility of an inverse association between the HDL-C/CRP ratio and chronic cough susceptibility. Prospective validation of this relationship, together with a mechanistic dissection of whether HDL-mediated CRP suppression operates locally within airways or systemically, represents important next steps.

### Strengths and limitations

This study confirmed a significant negative association between HDL-C/CRP ratio and chronic cough by controlling for confounders and constructing multiple models. This finding provides a new perspective for clinicians to understand the underlying inflammatory mechanisms of chronic cough.

This study has, however, several limitations. First, residual confounding may exist from unmeasured variables (air pollution, occupational dust) unavailable in NHANES. Second, missing data were excluded; if these data were not missing completely at random, this may introduce selection bias. Third, self-reported smoking and chronic cough may be subject to social desirability bias, leading to underreporting of smoking or respiratory symptoms. Fourth, single-time-point CRP and HDL-C measurements may not reflect long-term status due to biological variability. Fifth, the cross-sectional design precludes causal inference, and the temporal relationship between smoking exposure and dysregulation of the lipid-inflammation axis requires validation in prospective studies.

## CONCLUSIONS

This cross-sectional study found that the HDL-C/CRP ratio, a composite inflammatory marker, was associated with the risk of developing chronic cough. Specifically, a higher HDL-C/CRP ratio was associated with a lower risk of chronic cough. This ratio further provides new clues for understanding the inflammation-lipid mechanisms underlying chronic cough. Smoking status appears to modify the cross-sectional relationship between HDL-C/CRP ratio and chronic cough, with the inverse association most pronounced in never smokers. However, the clinical applicability of the HDL-C/CRP ratio in chronic cough disease needs to be validated by further prospective clinical trials.

## Supplementary Material



## Data Availability

The data supporting this research are publicly available datasets and are available from the following source: https://wwwn.cdc.gov/nchs/nhanes/.
