# Supplementary material for: Association of the high-density lipoprotein cholesterol to C-reactive protein ratio with chronic cough in US adults: Effect modification by smoking status
Source: Tob Induc Dis. 2026 Jul 26;24:10.18332/tid/222393. doi: 10.18332/tid/222393 (PMC13417970; doi:10.18332/tid/222393)
Supplement: Supplementary file 1 [file TID-24-124-s1.pdf]

TID
Tobacco Induced Diseases

Supplementary file

© 2026 Li X. et al.

DOI:
10.18332/tid/222393

The content has been provided by the author(s) and has not been reviewed, verified, or endorsed by European Publishing. It may not have undergone peer review. The views, opinions, and recommendations expressed are solely those of the author(s) and do not necessarily reflect the position of European Publishing. European Publishing accepts no responsibility or liability for any consequences arising from the use of, or reliance on, this content.

Supplementary Figure S4

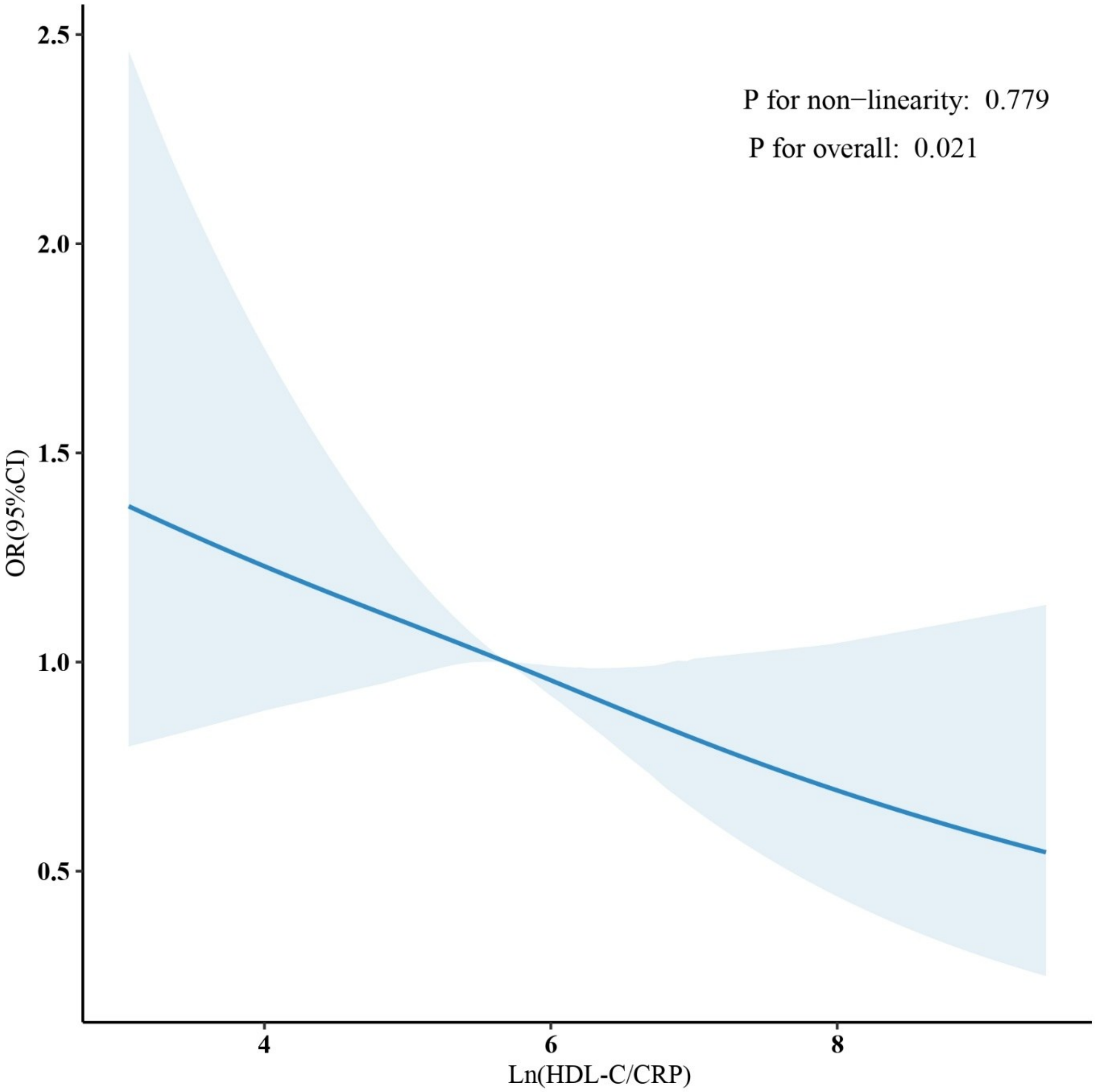

Note: Weighted restricted cubic spline analysis was conducted to examine the association between Ln (HDL-C/CRP) and chronic cough, adjusting for potential confounders including age, gender, race, BMI, education level, marital status, PIR, smoking status, drinking status, diabetes, asthma, emphysema, chronic bronchitis, cancer, heart failure, coronary artery disease, hypertension, P&I-30d, WBC, BEC, total cholesterol and stroke.

Abbreviations: Ln(HDL-C/CRP): natural logarithm of the ratio of high-density lipoprotein cholesterol to C-reactive protein; BMI: body mass index; PIR: poverty income ratio; P&I-30d: infections of pneumonia and influenza within the past 30 days; WBC: white blood cell count; BEC: blood eosinophil count.
